# DNA methylation age acceleration is associated with ALS age of onset and survival

**DOI:** 10.1007/s00401-020-02131-z

**Published:** 2020-03-07

**Authors:** Ming Zhang, Paul M. McKeever, Zhengrui Xi, Danielle Moreno, Christine Sato, Tessa Bergsma, Philip McGoldrick, Julia Keith, Janice Robertson, Lorne Zinman, Ekaterina Rogaeva

**Affiliations:** 1 Shanghai First Rehabilitation Hospital, School of Medicine, Tongji University, Shanghai, 200090 China; 2 Tanz Centre for Research in Neurodegenerative Diseases, University of Toronto, 60 Leonard Ave., Toronto, ON M5T 2S8 Canada; 3 Clinical Center for Brain and Spinal Cord Research, Tongji University, Shanghai, 200092 China; 4 Institute for Advanced Study, Tongji University, Shanghai, China; 5 Department of Laboratory Medicine and Pathobiology, University of Toronto, 27 King’s College Circle, Toronto, ON M5S 1A1 Canada; 6 Shanghai East Hospital, School of Medicine, Tongji University, Shanghai, 200120 China; 7 Sunnybrook Health Sciences Centre, 2075 Bayview Ave, Toronto, ON M4N 3M5 Canada; 8 Division of Neurology, Department of Medicine, University of Toronto, Toronto, Canada

Amyotrophic lateral sclerosis (ALS) patients, including *C9orf72*-carriers and identical twins [1], have highly variable disease characteristics (e.g., duration and age/site of onset) [7], suggesting the influence of epigenetic variations. DNA methylation (DNAm) is a key epigenetic modification and linked to the risk of several neurodegenerative diseases (Supplementary introduction). The cumulative assessment of DNAm levels at age-related CpGs allows the estimation of multi-tissue DNAm age (epigenetic clock), which could be more accurate for assessing biological age than chronological age. Hypermethylation of the CpG-island 5′ of the *C9orf72*-repeat and DNAm-age acceleration have been reported to be associated with *C9orf72*-disease duration and age of onset [9, 10]. However, epigenetic modifiers in genetically unexplained ALS patients are largely unknown.

Hence, we conducted a genome-wide investigation of DNAm (Infinium MethylationEPIC chip) in samples from blood (*n* = 249) or tissues from the central nervous system (CNS) (*n* = 18) of mainly sporadic ALS patients (Table 1), and assessed the association of DNAm-age acceleration with disease age of onset and survival (Supplementary methods). Considering the reported 3-year error for estimating DNAm age, we classified the patients into three groups: normal aging (*n* = 82, DNAm-age acceleration between − 3 and 3 years, median = 0.5 years), slow aging (*n* = 125, DNAm-age acceleration < − 3 years, median = − 6.3 years), and fast aging (*n* = 42, DNAm-age acceleration > 3 years, median = 5.7 years).

**Table 1:** Sample characteristics of the ALS cohort

Characteristics of ALS patients	Blood	CNS cohort^a^
Sporadic ALS cases (*n*)	200	17
Familial ALS cases (*n*)	49	1
Sex (male, *n* %)	148, 59.4%	13, 72.2%
Age of onset (years)		
Mean	59	59
Range	16–87	31–79
Site of onset (n, %)		
Bulbar	67, 27%	4, 22.2%
Limb	172, 73%	14, 77.8%

^a^Frontal cortex and cervical spinal cord from the central nervous system (CNS)

Multivariate linear regression analysis detected a highly significant association between blood-based DNAm-age acceleration and ALS age of onset (adjusted *p* value = 2.2E-16, *B* = − 1.29, *R*^2^ = 0.33, *n* = 249) (Supplementary methods, Fig. 1a), suggesting that for every 5-year increase in DNAm-age acceleration there is a 6.4-year earlier onset. Cox proportional hazard regression analysis revealed that a faster DNAm-age acceleration is significantly associated with an earlier age of onset [adjusted *p* value = 9.3E-16, hazard ratio (HR) = 2.12; 95% confidence interval (CI):1.76–2.54], suggesting that the hazard increased by 112% (Fig. 1c). The median age of onset in the fast aging group (48 years; 95%CI: 42–51) was 18 years younger than the slow aging group (66 years; 95%CI: 63–68). Significant results were not driven by subgroups stratified by sex or site of onset (Figs. S2–S3, Fig. S6).

**Fig. 1:**
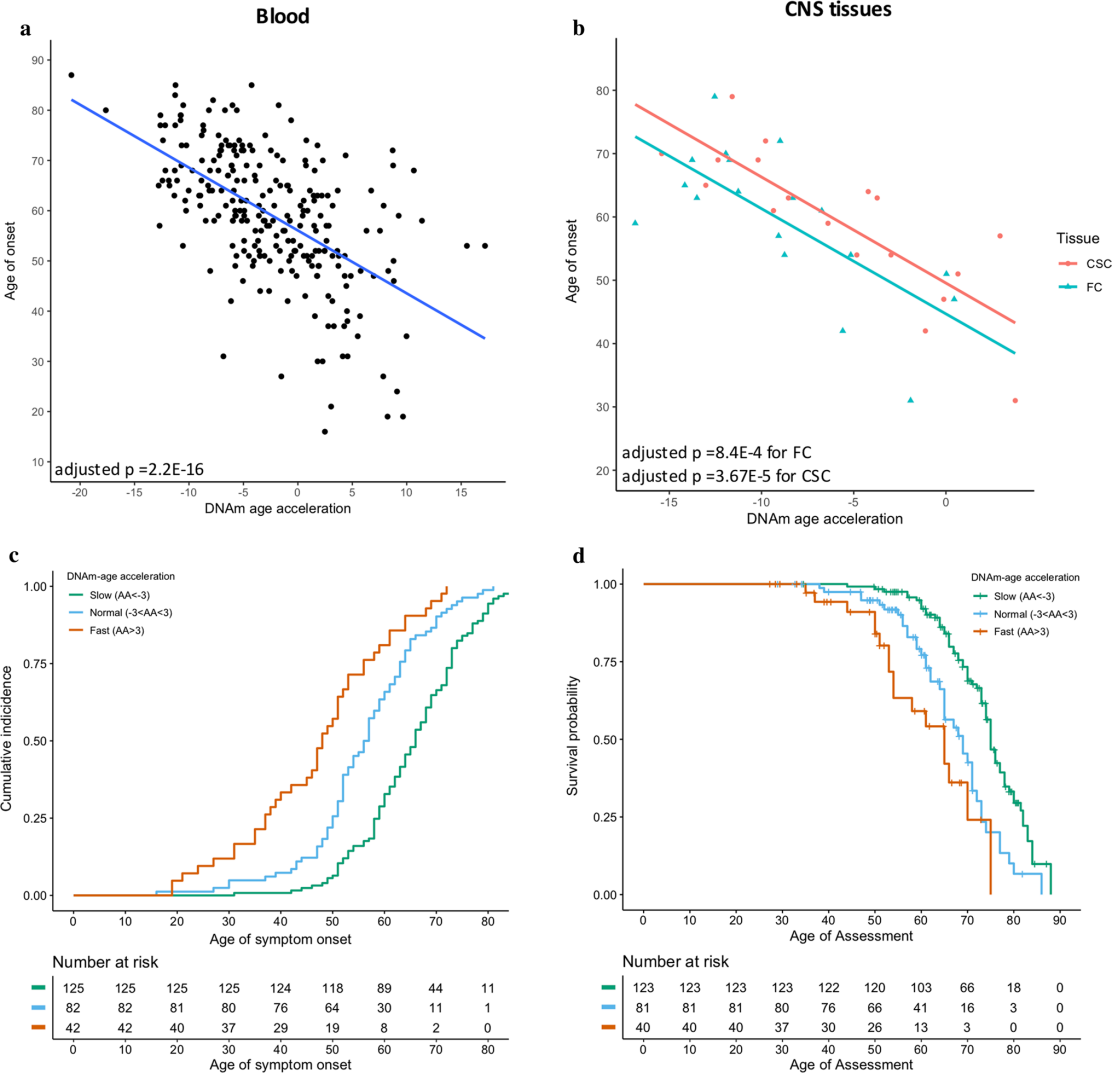
DNAm-age acceleration is significantly associated with ALS age of onset and survival. **a** Scatter plot showing the association between blood-based DNAm-age acceleration and age of onset (adjusted *p* value = 2.2E-16, *B* = − 1.29, *R*^2^ = 0.33; blood cell abundance adjusted *p* value = 2E-16, *B* = − 1.22, *R*^2^ = 0.47, *n* = 249). The solid line represents the linear regression trend. **b** Scatter plot showing the association between CNS-based DNAm-age acceleration and age of onset. Tissues were obtained from 18 ALS cases: frontal cortex (FC) (adjusted *p* value = 8.4E-4, *B* = − 1.73, *R*^2^ = 0.48), and cervical spinal cord (CSC) (adjusted *p* value = 3.67E-5, *B* = − 1.69, *R*^2^ = 0.65). The solid line represents the linear regression trend. **c** Kaplan–Meier curve of cumulative incidence of ALS age of onset (*n* = 249) stratified into three groups based on blood-based DNAm-age acceleration (adjusted *p* value = 9.3E-16, HR = 2.12; 95%CI: 1.76–2.54; blood cell abundance adjusted *p* value = 5.3E-12, HR = 2.39, 95%CI: 1.86–3.06). **d** Kaplan–Meier curve of survival probability (*n* = 244) stratified into three aging groups based on blood-based DNAm-age acceleration (adjusted *p* value = 1.7E-7, HR = 2.07; 95%CI: 1.58–2.72; blood cell abundance adjusted *p* value = 4.4E-8, HR = 2.21, 95%CI: 1.66–2.93). Slow aging: DNAm-age acceleration <  − 3 years, normal aging: DNAm-age acceleration between − 3 and 3 years, and fast aging: DNAm-age acceleration > 3 years. AA represents DNAm-age acceleration

Furthermore, Cox proportional hazard regression analysis revealed that a faster DNAm-age acceleration was significantly associated with a shorter survival with an increased hazard of 107% (adjusted *p* value = 1.7E-7, HR = 2.07; 95%CI: 1.58–2.72) (Supplementary methods, Fig. 1d). The median survival age was 10 years shorter in the fast aging group (65 years; 95%CI: 54–70) than the slow aging group (75 years; 95%CI: 74–77). We replicated this observation by re-analyzing DNAm data from our previous study of 30 *C9orf72 *patients [10], and found that the median survival age was 15 years shorter in the fast vs slow aging group (adjusted *p* value = 0.01, HR = 3.5; 95%CI: 1.3–9.3), suggesting that the hazard increased by 250% (Fig. S1). The significant findings were not driven by subgroups stratified by sex or site of onset (Fig. S4–S6).

Previously, we reported that CNS-based DNAm-age acceleration is significantly associated with *C9orf72-*disease age of onset and duration, using DNA from frontal cortex (FC) and cervical spinal cord (CSC) [10]. To evaluate this association in genetically unexplained ALS patients, we investigated DNA isolated from FC and CSC tissues of 18 ALS cases. Even with the modest CNS sample size, DNAm-age acceleration is associated with age of onset in DNA from FC (adjusted *p* value = 8.4E-4, B = − 1.73, *R*^2^ = 0.48); and CSC (adjusted *p* value = 3.7E-5, B = − 1.69, *R*^2^ = 0.65) (Fig. 1b). The linear regression model suggests that a 5-year increase in CNS-based DNAm-age acceleration is linked to an 8.4–8.7 year earlier onset. Furthermore, the Cox proportional hazard regression analysis suggests that CNS-based DNAm-age acceleration is significantly associated with survival (FC: *p* value = 0.006; CSC: *p* value = 0.0009) (Fig. S7). As expected for a multi-tissue epigenetic clock, there was no significant difference in DNAm-age acceleration between the two CNS tissues (*p* value = 0.13; Fig. S8). Since ALS is often associated with mixed pathologies [2], we evaluated if FC pathology (tau or TDP-43 inclusions, Table S1) may affect our findings. However, a subgroup analysis of patients with (*n* = 5) or without (*n* = 13) FC pathology found no significant difference in DNAm-age acceleration (*p* value = 0.78, Fig. S9).

Locus-by-locus analysis of blood DNAm levels of 835,424 CpGs (not overlapping known genetic polymorphisms) found no CpGs associated with age of onset at the genome-wide significant level in 249 ALS patients (Fig. S10, Table S2–S3).

Cumulatively, our study revealed that blood/CNS-based DNAm-age acceleration is significantly associated with age of onset and survival in genetically unexplained ALS patients, suggesting a novel epigenetic modifier. In *C9orf72* patients, we observed a trend for a stronger association of survival with DNAm-age acceleration vs a general ALS cohort, but with a much broader 95%CI (likely due to the modest size of the *C9orf72* cohort; *n* = 30). DNAm-age acceleration was comparable between the two investigated CNS tissues; however, we observed a tendency for a stronger association of DNAm-age acceleration with age of onset in CNS vs non-autopsy blood samples. Both trends should be clarified by analyzing a larger sample set in the future (Supplementary discussion).

Our findings strongly support the use of DNAm-age acceleration as a biomarker of biological aging. The epigenetic clock may also be used to stratify results obtained in clinical trials (e.g., response to treatment in the fast vs slow aging groups). Notably, a recent pilot study reported using the epigenetic clock in a clinical trial intended to regenerate the thymus to prevent signs of immunosenescence [3], encouraging the use of the epigenetic clock to estimate the effectiveness of aging interventions.

The molecular mechanism underlying the epigenetic clock and its link to disease presentation is largely unknown. Age-related CpGs are mapped to promoter, enhancer and polycomb protein-binding regions [4]. Aberrant DNAm in these regions may regulate gene expression involved in key biological processes (cell growth/proliferation and death/survival [5]). Future studies could also clarify the association between DNAm-age acceleration and other ALS phenotypes (e.g., severity), or in ALS patients with other mutations (e.g., in *SOD1*), and different neurodegenerative phenotypes in the longitudinal Genetic Frontotemporal dementia Initiative cohort [6] and the Dominantly inherited Alzheimer’s disease cohort [8] (Supplementary discussion).

Hence, DNAm-age acceleration may be used as an aging biomarker in future clinical trials to predict the effect of drugs on modulating ALS age of onset or survival.

## Electronic supplementary material

Below is the link to the electronic supplementary material.

Supplementary file1 (PDF 3758 kb)
