# Supplementary material for: DNA methylation age acceleration is associated with ALS age of onset and survival
Source: Acta Neuropathol. 2020 Mar 7;139(5):943–6. doi: 10.1007/s00401-020-02131-z (PMC7181538; doi:10.1007/s00401-020-02131-z)
Supplement: Supplementary file 1 — Supplementary file1 (PDF 3758 kb) [file 401_2020_2131_MOESM1_ESM.pdf]

Electronic supplementary materials

DNA methylation age acceleration is associated with ALS age of onset and survival

Ming Zhang, PhD[+1-4], Paul M. McKeever, PhD[2,5], Zhengrui Xi, PhD[3,6], Danielle Moreno, BSc[2], Christine Sato, MSc[2], Tessa Bergsma[2], Philip McGoldrick, PhD[2,5], Julia Keith, MD[5,7], Janice Robertson, PhD[2,5], Lorne Zinman, MD[+7,8], and Ekaterina Rogaeva, PhD[+2,8]

[1] Shanghai First Rehabilitation Hospital, School of Medicine, Tongji University, 200090, Shanghai, China
[2] Tanz Centre for Research in Neurodegenerative Diseases, University of Toronto, 60 Leonard Ave., Toronto, ON, Canada, M5T 2S8
[3] Clinical Center for Brain and Spinal Cord Research, Tongji University, 200092, Shanghai, China
[4] Institute for Advanced Study, Tongji University, Shanghai, China
[5] Department of Laboratory Medicine and Pathobiology, University of Toronto, 27 King's College Circle, Toronto, ON, Canada, M5S 1A1
[6] Shanghai East Hospital, School of Medicine, Tongji University, 200120, Shanghai, China
[7] Sunnybrook Health Sciences Centre, 2075 Bayview Ave., Toronto, ON, Canada, M4N 3M5
[8] Division of Neurology, Department of Medicine, University of Toronto, Toronto, Canada

+ Correspondence should be addressed to:
Ekaterina Rogaeva (ekaterina.rogaeva@utoronto.ca)
Tanz Centre for Neurodegenerative Diseases, 60 Leonard Avenue, Toronto, ON, Canada, M5T 2S8; tel: (416)507-6872; Fax: (416) 603-6435
or
Lorne Zinman (Lorne.Zinman@sunnybrook.ca)
Sunnybrook Health Sciences Centre, 2075 Bayview Ave., Toronto, ON, M4N 3M5
tel: 416-480-4213; Fax: 416-480-6817
or
Ming Zhang (mingzhang@tongji.edu.cn)
Shanghai First Rehabilitation Hospital, School of Medicine, Tongji University, Shanghai, China, 200090

Supplementary introduction

High phenotypic heterogeneity has been observed even in ALS patients carrying the same Mendelian mutation [8]. For example, patients with a G_4C_2-expansion in *C9orf72* have variable disease duration (0.5-22 years) and age of onset (27-74 years) [4]. Furthermore, high discordance in age of onset was reported in identical twins [1], including Canadian twin pairs carrying either a *SOD1* substitution or *C9orf72* G_4C_2-expansion [20, 26]. Epigenetic variations that bridge genetic and environmental factors [18] may contribute to variable disease survival and age of onset in ALS patients.

DNA methylation (DNAm) at CpG dinucleotides is one of the key epigenetic modifications modulating gene expression or splicing. Prior locus-by-locus DNAm studies have suggested several susceptibility genes with potential differential expression/splicing effects in neurodegenerative diseases, including Alzheimer's disease [5, 23], Huntington's disease [19], dementia with Lewy bodies and Parkinson's disease [16]. There are only a few DNAm studies in ALS. For instance, it was shown that hypermethylation of the CpG-island 5' of the G_4C_2-repeat in *C9orf72* is associated with a longer disease duration in *C9orf72* ALS patients [21]. Furthermore, the *C9orf72* repeat size may affect age at onset by regulating DNAm levels at this 5' CpG-island [7].

Importantly, DNAm is closely linked to aging, which is the strongest risk factor for ALS and other neurodegenerative disorders. The DNAm levels at 353 CpGs on the 450K BeadChip were reported to be age-related, and their cumulative assessment allows for the estimation of DNAm-age, which is an accurate predictor of chronological age across different tissues, including blood and brain (R=0.96) [9]. Notably, DNAm-age could be more precise for estimating biological age than chronological age. Indeed, we previously revealed that a discrepancy between DNAm-age and chronological age (DNAm-age acceleration) is significantly associated with disease age of onset and duration in *C9orf72* patients, making it a potential biomarker of biological aging (for every 5-year increase in DNAm age-acceleration we observed a 3.2-year earlier

age of onset and 1.5-year shorter disease duration) [25]. Furthermore, studies of ALS-discordant identical twin pairs revealed an accelerated DNAm-age in the affected twin compared to the unaffected twin [17, 22, 26]. In addition, DNAm-age acceleration has been found to be significantly associated with mortality [13], and several neurodegenerative diseases (e.g. Parkinson's disease [11], Huntington's disease [10] and Alzheimer's disease [12, 14, 23]). However, it is unknown if DNAm-age acceleration is a modifier of age of onset or survival in a more general mainly sporadic cohort of ALS patients without known mutations.

Supplementary methods

Human samples

Informed consent was obtained from all participants in accordance with the ethics review boards at Sunnybrook Health Sciences Centre and University of Toronto. The study included 49 familial and 200 sporadic Canadian ALS patients of Caucasian ethnicity without causal mutations in *C9orf72, SOD1, TARDBP, FUS,* or *CHCHD10*. Blood samples were collected at the Sunnybrook Health Sciences Centre ALS Clinic at the time of ALS diagnosis, which was done based on the El Escorial revisited clinical criteria [2]. Age of onset was self-reported and defined as the age at which the first bulbar or limb symptom appeared. Survival status was available for 244 patients and calculated as reported previously [6] (the difference between age at last follow-up and age of onset for 123 patients with ongoing disease; or age at death and age of onset for 121 deceased patients). The neuropathological diagnosis for 18 autopsy ALS cases and findings in the frontal cortex and cervical spinal cord are provided in Table S1.

Epigenetic analyses

DNA samples were bisulfite converted using the EZ DNA Methylation-Lightning™ Kit (Zymo). DNAm levels were analyzed using the genome-wide Infinium MethylationEPIC chip covering ~850,000 CpGs. The β-value was used to estimate the DNAm level of each CpG using the intensity

ratio between the methylated and unmethylated alleles (β-value of 0: non-methylated; β-value of 1: completely methylated). After CpG-sites overlapping known single nucleotide polymorphisms were removed; 835,424 CpGs were included in the analysis.

We used the DNAm-age calculator (https://dnamage.genetics.ucla.edu/) to determine DNAm-age based on the DNAm levels of 334 CpGs on the Infinium MethylationEPIC chip, which includes 90% of the 353 age-related CpGs from the discontinued 450K BeadChip, providing a similar capacity to estimate DNAm-age as the 450K BeadChip [15]. DNAm-age acceleration was calculated as DNAm-age minus chronological age at date of sample collection. In addition, we used the advanced analysis mode of the DNAm-age calculator to estimate blood cell abundance [9] for plasma blasts, CD8+CD28-CD45RA- T cells and naive CD8 T cells, which were adjusted for in the multivariate linear regression and Cox proportional hazard regression analyses.

Statistics

We used a Cox proportional hazard regression model (R survival and survminer packages) [24] adjusting for sex, site of onset, and censoring age at last follow-up for the 123 ALS patients with ongoing disease (for the survival analysis only). The hazard ratio (HR) with 95% confidence interval (CI) is presented. The Kaplan-Meier estimate was used to obtain the median age of onset or survival for the three aging groups: normal aging (n=82, DNAm-age acceleration between -3 and 3 years, median=0.5 years), slow aging (n=125, DNAm-age acceleration <-3 years, median=-6.3 years), and fast aging (n=42, DNAm-age acceleration >3 years, median=5.7 years). Multivariate linear regression was used to analyze the association of DNAm-age acceleration with age of onset [25], and obtain p-values adjusted for sex. We presented the linear regression coefficient (B) with standard error (SE) and percentage of response variance explained by the linear regression model (R^2). We also adjusted p-values to blood cell abundance for both multivariate regression and Cox proportional hazard regression analysis when appropriate. The Mann Whitney U (MWU) test was used to compare the mean difference in DNAm-age acceleration or age of onset.

We used the R minfi package to analyze the genome-wide DNAm data [24]. Multivariate linear regression was used to estimate the association between locus-by-locus DNAm changes and age of onset adjusted for age at sample collection, sex, and site of onset (q-values represent false discovery rate). We also applied a Bonferroni correction to adjust for multiple comparisons at the genome-wide level (adjusted p-values<6.0E-8 were accepted as statistically significant). R-project 3.3.1 was used for the statistical analysis. The R qqman package was used for generating the Manhattan plot [24].

Supplementary discussion

Our study revealed that increased DNAm-age acceleration is significantly associated with an earlier age of onset and shorter survival in genetically unexplained ALS patients. A faster DNAm-age acceleration is significantly associated with a shorter survival with an increased hazard of 107% (supplementary methods, Fig. 1d). A similar trend was detected by re-analyzing DNAm data from our previous study of *C9orf72*-ALS patients [25], suggesting hazard increased by 250% (Fig. S1). However, we observed a much broader 95%CI in the *C9orf72* cohort (1.3-9.3) vs general ALS patients (1.6-2.7), which is probably due to the modest number of *C9orf72* patients (n=30). In patients carrying the same genetic mutation (e.g. *C9orf72* G_4C_2-expansion), it might be easier to detect the effect of epigenetic/genetic modifiers due to reduced heterogeneity. For example, in our previous study [24], we observed that the rs9357140 GG-genotype had a greater protective effect in *C9orf72* patients compared to *C9orf72* negative patients. More ALS samples with or without known mutations would be needed to validate our findings in future studies. For instance, it would be important to evaluate the link between DNAm-age acceleration and ALS presentations in carriers of other causal mutations (e.g. in *SOD1*). Future studies could also investigate if DNAm-age acceleration is linked to other phenotypes (e.g. ALS severity characterized by the revised ALS functional rating scale: ALSFRS-R [3], or age at onset in the Genetic Frontotemporal dementia Initiative (GENFI) cohort and the Dominantly inherited Alzheimer's disease cohort.

Previous studies have suggested that DNAm-age reflects aging status similarly in different tissues, except for sperm, cerebellum [25] and breast [9]. In the current study, we observed a similar correlation of DNAm-age acceleration with age of onset in blood (n=249) and frontal cortex or cervical spinal cord tissues (n=18) (Fig. 1). No significant difference in DNAm-age acceleration was observed between the two investigated CNS tissues (Fig. S8). Of note, we found that a 5-year increase in DNAm-age acceleration was linked to an 8.4-8.7 year earlier onset using DNA from CNS tissues vs a 6.4-year earlier onset using DNA from blood collected at ALS diagnosis. The observed ~2-year difference could reflect disease progression, indicating further acceleration of DNAm-age by the disease end-stage. However, this result has to be interpreted with caution due to the modest size of the CNS cohort, and the fact that the DNAm-age estimator has an error of ~3 years [9]. Future studies should investigate both blood and CNS tissues collected at the same time-point (at autopsy) to evaluate the stability of DNAm-age acceleration as an aging biomarker and assess its utility for clinical trials.

Supplementary reference

1 Al-Chalabi A, Fang F, Hanby MF, Leigh PN, Shaw CE, Ye W, Rijsdijk F (2010) An estimate of amyotrophic lateral sclerosis heritability using twin data. J Neurol Neurosurg Psychiatry 81: 1324-1326 Doi 10.1136/jnnp.2010.207464

2 Brooks BR, Miller RG, Swash M, Munsat TL, World Federation of Neurology Research Group on Motor Neuron D (2000) El Escorial revisited: revised criteria for the diagnosis of amyotrophic lateral sclerosis. Amyotroph Lateral Scler Other Motor Neuron Disord 1: 293-299 Doi 10.1080/146608200300079536

3 Cedarbaum JM, Stambler N, Malta E, Fuller C, Hilt D, Thurmond B, Nakanishi A (1999) The ALSFRS-R: a revised ALS functional rating scale that incorporates assessments of respiratory function. BDNF ALS Study Group (Phase III). J Neurol Sci 169: 13-21 Doi 10.1016/s0022-510x(99)00210-5

4 Cooper-Knock J, Hewitt C, Highley JR, Brockington A, Milano A, Man S, Martindale J, Hartley J, Walsh T, Gelsthorpe Cet al (2012) Clinico-pathological features in amyotrophic lateral sclerosis with expansions in C9ORF72. Brain 135: 751-764 Doi 10.1093/brain/awr365

5 De Jager PL, Srivastava G, Lunnon K, Burgess J, Schalkwyk LC, Yu L, Eaton ML, Keenan BT, Ernst J, McCabe Cet al (2014) Alzheimer's disease: early alterations in brain DNA methylation at ANK1, BIN1, RHBDF2 and other loci. Nat Neurosci 17: 1156-1163 Doi 10.1038/nn.3786
6 Fogh I, Lin K, Tiloca C, Rooney J, Gellera C, Diekstra FP, Ratti A, Shatunov A, van Es MA, Proitsi Pet al (2016) Association of a Locus in the CAMTA1 Gene With Survival in Patients With Sporadic Amyotrophic Lateral Sclerosis. JAMA Neurol 73: 812-820 Doi 10.1001/jamaneurol.2016.1114
7 Gijselinck I, Van Mossevelde S, van der Zee J, Sieben A, Engelborghs S, De Bleecker J, Ivanoiu A, Deryck O, Edbauer D, Zhang Met al (2016) The C9orf72 repeat size correlates with onset age of disease, DNA methylation and transcriptional downregulation of the promoter. Mol Psychiatry 21: 1112-1124 Doi 10.1038/mp.2015.159
8 Hardy J, Rogaeva E (2014) Motor neuron disease and frontotemporal dementia: sometimes related, sometimes not. Exp Neurol 262 Pt B: 75-83 Doi 10.1016/j.expneurol.2013.11.006
9 Horvath S (2013) DNA methylation age of human tissues and cell types. Genome Biol 14: R115 Doi 10.1186/gb-2013-14-10-r115
10 Horvath S, Langfelder P, Kwak S, Aaronson J, Rosinski J, Vogt TF, Eszes M, Faull RL, Curtis MA, Waldvogel HJet al (2016) Huntington's disease accelerates epigenetic aging of human brain and disrupts DNA methylation levels. Aging 8: 1485-1512 Doi 10.18632/aging.101005
11 Horvath S, Ritz BR (2015) Increased epigenetic age and granulocyte counts in the blood of Parkinson's disease patients. Aging 7: 1130-1142 Doi 10.18632/aging.100859
12 Levine ME, Lu AT, Bennett DA, Horvath S (2015) Epigenetic age of the pre-frontal cortex is associated with neuritic plaques, amyloid load, and Alzheimer's disease related cognitive functioning. Aging 7: 1198-1211 Doi 10.18632/aging.100864
13 Marioni RE, Shah S, McRae AF, Chen BH, Colicino E, Harris SE, Gibson J, Henders AK, Redmond P, Cox SRet al (2015) DNA methylation age of blood predicts all-cause mortality in later life. Genome Biol 16: 25 Doi 10.1186/s13059-015-0584-6
14 McCartney DL, Stevenson AJ, Walker RM, Gibson J, Morris SW, Campbell A, Murray AD, Whalley HC, Porteous DJ, McIntosh AMet al (2018) Investigating the relationship between DNA methylation age acceleration and risk factors for Alzheimer's disease. Alzheimers Dement (Amst) 10: 429-437 Doi 10.1016/j.dadm.2018.05.006
15 McEwen LM, Jones MJ, Lin DTS, Edgar RD, Husquin LT, MacIsaac JL, Ramadori KE, Morin AM, Rider CF, Carlsten Cet al (2018) Systematic evaluation of DNA methylation age estimation with common preprocessing methods and the Infinium MethylationEPIC BeadChip array. Clin Epigenetics 10: 123 Doi 10.1186/s13148-018-0556-2
16 Sanchez-Mut JV, Heyn H, Vidal E, Moran S, Sayols S, Delgado-Morales R, Schultz MD, Ansoleaga B, Garcia-Esparcia P, Pons-Espinal Met al (2016) Human DNA methylomes of neurodegenerative diseases show common epigenomic patterns. Transl Psychiatry 6: e718 Doi 10.1038/tp.2015.214

17 Tarr IS, McCann EP, Benyamin B, Peters TJ, Twine NA, Zhang KY, Zhao Q, Zhang ZH, Rowe DB, Nicholson GAet al (2019) Monozygotic twins and triplets discordant for amyotrophic lateral sclerosis display differential methylation and gene expression. Sci Rep 9: 8254 Doi 10.1038/s41598-019-44765-4
18 Taudt A, Colome-Tatche M, Johannes F (2016) Genetic sources of population epigenomic variation. Nat Rev Genet 17: 319-332 Doi 10.1038/nrg.2016.45
19 Wood H (2013) Neurodegenerative disease: altered DNA methylation and RNA splicing could be key mechanisms in Huntington disease. Nat Rev Neurol 9: 119 Doi 10.1038/nrneurol.2013.23
20 Xi Z, Yunusova Y, van Blitterswijk M, Dib S, Ghani M, Moreno D, Sato C, Liang Y, Singleton A, Robertson Jet al (2014) Identical twins with the C9orf72 repeat expansion are discordant for ALS. Neurology 83: 1476-1478 Doi 10.1212/WNL.0000000000000886
21 Xi Z, Zinman L, Moreno D, Schymick J, Liang Y, Sato C, Zheng Y, Ghani M, Dib S, Keith Jet al (2013) Hypermethylation of the CpG island near the G4C2 repeat in ALS with a C9orf72 expansion. Am J Hum Genet 92: 981-989 Doi 10.1016/j.ajhg.2013.04.017
22 Young PE, Kum Jew S, Buckland ME, Pamphlett R, Suter CM (2017) Epigenetic differences between monozygotic twins discordant for amyotrophic lateral sclerosis (ALS) provide clues to disease pathogenesis. PLoS One 12: e0182638 Doi 10.1371/journal.pone.0182638
23 Zhang M, Dilliott AA, Khallaf R, Robinson JF, Hegele RA, Comishen M, Sato C, Tosto G, Reitz C, Mayeux Ret al (2019) Genetic and epigenetic study of an Alzheimer's disease family with monozygotic triplets. Brain 142: 3375-3381 Doi 10.1093/brain/awz289
24 Zhang M, Ferrari R, Tartaglia MC, Keith J, Surace EI, Wolf U, Sato C, Grinberg M, Liang Y, Xi Zet al (2018) A C6orf10/LOC101929163 locus is associated with age of onset in C9orf72 carriers. Brain 141: 2895-2907 Doi 10.1093/brain/awy238
25 Zhang M, Tartaglia MC, Moreno D, Sato C, McKeever P, Weichert A, Keith J, Robertson J, Zinman L, Rogaeva E (2017) DNA methylation age-acceleration is associated with disease duration and age at onset in C9orf72 patients. Acta Neuropathol 134: 271-279 Doi 10.1007/s00401-017-1713-y
26 Zhang M, Xi Z, Ghani M, Jia P, Pal M, Werynska K, Moreno D, Sato C, Liang Y, Robertson Jet al (2016) Genetic and epigenetic study of ALS-discordant identical twins with double mutations in SOD1 and ARHGEF28. J Neurol Neurosurg Psychiatry 87: 1268-1270 Doi 10.1136/jnnp-2016-313592

Table S1. Sample characteristics and pathology of the 18 ALS patients with frontal cortex and cervical spinal cord tissue.

CRND ID	Age of onset	Age at autopsy	Sex	Family history of ALS	Clinical diagnosis	Pathological diagnosis*	FC pathology	CSC pathology
7545	61	64	Male	No	ALS	ALS	none	TDP43
8363	69	70	Male	No	ALS	ALS	none	TDP43
8613	42	43	Female	No	ALS	ALS	none	TDP43
8756	64	65	Male	No	ALS	ALS + mild AD	rare tau inclusions	TDP43
8866	69	74	Female	No	ALS	ALS	none	TDP43
8875	79	83	Male	No	ALS	ALS + mild AD	rare tau inclusions	TDP43
8876	54	57	Male	No	ALS	ALS	none	TDP43
9465	31	37	Male	No	ALS	ALS	none	TDP43
9475	63	71	Female	No	ALS	ALS/FTLD	TDP43	TDP43
9481	63	66	Male	No	ALS	ALS	none	TDP43
9525	54	57	Female	No	ALS	ALS	none	TDP43
9703	57	61	Male	No	ALS	ALS	none	TDP43
9710	59	68	Female	Yes	ALS	ALS	none	TDP43
9772	72	75	Male	No	ALS	ALS	none	TDP43
10121	47	50	Male	No	ALS	ALS + MS	none	TDP43
10137	51	55	Male	No	ALS	ALS/FTLD	TDP43	TDP43
10221	70	73	Male	No	ALS	ALS + early AD	none	TDP43
10520	65	73	Male	No	ALS	ALS/FTLD-tau with PSP	rare tau inclusions	TDP43 + tau inclusions

ALS = amyotrophic lateral sclerosis; FTLD = frontal and temporal lobar degeneration; AD = Alzheimer's disease; MS = multiple sclerosis; PSP = progressive supranuclear palsy; FC = frontal cortex; CSC = cervical spinal cord

* Pathological diagnosis based on routine immunohistochemical analysis from staining with Hematoxylin&Eosin/Luxol Fast Blue, and antibodies against TDP43, p62, tau (AT8), beta-amyloid, alpha-synuclein, and/or neurofilament

Table S2. Top 20 nominally significant CpG-sites associated with age of ALS onset.

ID	Chromosome	Location (GRCh37/hg19)	Gene	Adjusted p-value *
cg10354512	8	134202337	*WISP1*	7.8E-08
cg12043747	11	75272845	*SERPINH1*	9.8E-08
cg19327844	11	6440482	*APBB1*	1.03E-07
cg11133774	17	47629554	*LOC100288866*	1.13E-07
cg09263990	1	2115621	*PRKCZ*	1.2E-07
cg20349687	2	88927127	*EIF2AK3*	1.4E-07
cg13024068	18	44269268	*ST8SIA5*	1.5E-07
cg10810026	8	118894613	*EXT1*	1.6E-07
cg07736658	2	121105175	*INHBB*	1.7E-07
cg10133777	19	36005906	*DMKN*	1.8E-07
cg16034060	19	4953233	*UHRF1*	1.9E-07
cg02204965	19	6205253		2.1-07
cg07812904	16	57307617	*PLLP*	2.5E-07
cg20301680	1	54786494	*SSBP3*	2.5E-07
cg07955881	11	44639397	*CD82*	2.5E-07
cg21354621	12	46384284	*SFRS2IP*	2.6E-07
cg15884880	3	187764161		2.7E-07
cg07589824	1	86571125	*COL24A1*	2.8E-07
cg22341273	8	145234727	*HEATR7A*	3.0E-07
cg04985251	3	119188494	*KTELC1*	3.1E-07

* adjusted for age at sample collection, sex and site of onset.

Table S3. Association between the DNAm level of 82 DNAm-age related CpG-sites (q<0.05) and age of onset. None of the 334 age-related CpGs are associated with age of onset after correction for multiple testing and adjustment for age at sample collection, sex and site of onset.

ID	Gene	pval	qval	beta range	adjusted p-value
**cg04474832**	*ABHD14A*	7.10E-14	3.45E-11	0.27	0.69
**cg18328933**	*ABHD14A*	0.006685	0.041746	0.22	0.83
**cg03330058**	*ABTB1*	0.000073	0.001481	0.36	0.67
**cg11314684**	*AKT3*	0.000152	0.002620	0.31	0.13
**cg07730301**	*ALDH3B1*	0.000119	0.002167	0.44	0.84
**cg22947000**	*BCMO1*	5.16E-12	1.51E-09	0.43	0.05
**cg13547237**	*Bles03*	0.000306	0.004476	0.31	0.34
**cg27169020**	*BNC1*	0.001571	0.015131	0.35	0.45
**cg21801378**	*BRUNOL6*	4.10E-12	1.24E-09	0.43	0.82
**cg01560871**	*C10orf27*	0.000001	0.000032	0.31	0.46
**cg04126866**	*C10orf99*	0.000001	0.000063	0.23	0.60
**cg26005082**	*C19orf30*	1.26E-07	8.71E-06	0.27	0.98
**cg10865119**	*C6orf208*	0.000001	0.000037	0.24	0.85
**cg01353448**	*C7orf16*	0.000853	0.009683	0.47	0.59
**cg23124451**	*CBX7*	1.20E-19	2.76E-16	0.21	0.72
**cg19724470**	*CD274*	1.64E-10	3.07E-08	0.32	0.39
**cg17655614**	*CDH1*	0.000003	0.000109	0.30	0.72
**cg12373771**	*CECR6*	5.41E-13	2.08E-10	0.35	0.54
**cg14163776**	*CENTB2*	0.001204	0.012478	0.21	0.14
**cg20761322**	*CIB2*	0.000212	0.003380	0.48	0.02
**cg00168942**	*CX40.1*	0.000001	0.000029	0.28	0.07

**cg19692710**	*DNAJB13*	0.000161	0.002741	0.31	0.71
**cg04836038**	*DOCK9*	1.82E-07	1.18E-05	0.32	0.77
**cg01027739**	*DOLPP1*	0.000290	0.004299	0.30	0.87
**cg13460409**	*DSCR6*	0.000001	0.000033	0.43	0.60
**cg09809672**	*EDARADD*	1.82E-07	1.18E-05	0.39	0.78
**cg17274064**	*ERG*	0.000362	0.005092	0.24	0.83
**cg24888049**	*FES*	0.003133	0.024792	0.41	0.33
**cg16547529**	*FLJ33790*	0.002464	0.020917	0.40	0.40
**cg07158339**	*FXN*	6.09E-09	6.81E-07	0.33	0.21
**cg24058132**	*GALC*	0.000001	0.000059	0.26	0.29
**cg20914508**	*GAP43*	0.002663	0.022099	0.21	0.40
**cg21870884**	*GPR25*	2.64E-08	2.35E-06	0.31	0.17
**cg25148589**	*GRIA2*	4.62E-11	1.02E-08	0.31	0.25
**cg25771195**	*GTL3*	7.56E-08	5.67E-06	0.37	0.87
**cg25809905**	*ITGA2B*	3.88E-09	4.64E-07	0.43	0.03
**cg18573383**	*KCNC2*	2.92E-08	2.56E-06	0.30	0.65
**cg05675373**	*KCNC4*	0.000113	0.002085	0.58	0.70
**cg17729667**	*KIAA0980*	0.000058	0.001239	0.31	0.05
**cg04528819**	*KLF14*	1.79E-12	5.96E-10	0.34	0.46
**cg26842024**	*KLF2*	0.005338	0.035867	0.24	0.99
**cg05294243**	*KLK13*	0.000002	0.000072	0.21	0.89
**cg25564800**	*KPNA1*	0.000042	0.000952	0.37	0.52
**cg01820374**	*LAG3*	3.23E-20	8.62E-17	0.28	0.21
**cg03578041**	*LARP6*	0.003229	0.025325	0.37	0.53
**cg15804973**	*MAP3K5*	7.37E-22	3.21E-18	0.33	0.25

**cg11299964**	*MAPKAP1*	0.000002	0.000070	0.30	0.02
**cg14423778**	*MBNL1*	0.000052	0.001130	0.35	0.74
**cg14308452**	*MGC24975*	0.002192	0.019227	0.34	0.71
**cg27015931**	*MGC50721*	0.000054	0.001165	0.27	0.71
**cg13302154**	*MGP*	0.000036	0.000845	0.37	0.85
**cg13828047**	*MPI*	3.13E-08	2.71E-06	0.24	0.58
**cg07388493**	*NDUFS5*	5.92E-17	6.91E-14	0.35	0.99
**cg22736354**	*NHLRC1*	4.28E-25	4.25E-21	0.25	0.86
**cg08370996**	*NR2F2*	8.16E-10	1.23E-07	0.29	0.16
**cg05442902**	*P2RXL1*	0.000009	0.000283	0.51	0.31
**cg09418283**	*PAWR*	0.000001	0.000039	0.38	0.10
**cg12941369**	*PDCD6IP*	0.000001	0.000029	0.28	0.63
**cg13899108**	*PDE4C*	0.000042	0.000957	0.48	0.74
**cg07408456**	*PGLYRP2*	1.71E-08	1.63E-06	0.32	0.67
**cg20240860**	*PHACS*	0.000007	0.000241	0.23	0.32
**cg16744741**	*PRKG2*	4.69E-13	1.84E-10	0.25	0.80
**cg17324128**	*RASSF4*	0.000109	0.002031	0.24	0.48
**cg24450312**	*RASSF5*	0.003467	0.026618	0.32	0.37
**cg01968178**	*REEP1*	0.000856	0.009705	0.33	0.52
**cg10523019**	*RHBDD1*	0.000289	0.004280	0.35	0.00
**cg22809047**	*RPL31*	4.82E-11	1.06E-08	0.30	0.98
**cg26614073**	*SCAP*	2.17E-18	3.64E-15	0.39	0.90
**cg06493994**	*SCGN*	4.24E-26	5.30E-22	0.22	0.56
**cg01459453**	*SELP*	0.000002	0.000067	0.35	0.02
**cg02388150**	*SFRP1*	0.000146	0.002543	0.34	0.69

**cg17589341**	*SLC14A1*	0.000088	0.001718	0.33	0.36
**cg10345936**	*SLC36A2*	0.002370	0.020338	0.24	0.36
**cg08331960**	*SLC9A3R2*	0.000006	0.000209	0.32	0.43
**cg22679120**	*SNX8*	0.006057	0.039081	0.28	0.44
**cg26845300**	*SNX9*	0.002364	0.020302	0.60	0.16
**cg01511567**	*SSRP1*	1.38E-11	3.56E-09	0.22	0.81
**cg13836627**	*TJP1*	0.000632	0.007749	0.23	0.41
**cg23517605**	*TUBB2B*	0.007308	0.044344	0.38	0.79
**cg04084157**	*VGF*	0.000005	0.000172	0.27	0.83
**cg02071305**	*VPS18*	8.38E-08	6.19E-06	0.27	0.41
**cg14654875**	*ZNF597*	0.003072	0.024448	0.22	0.55

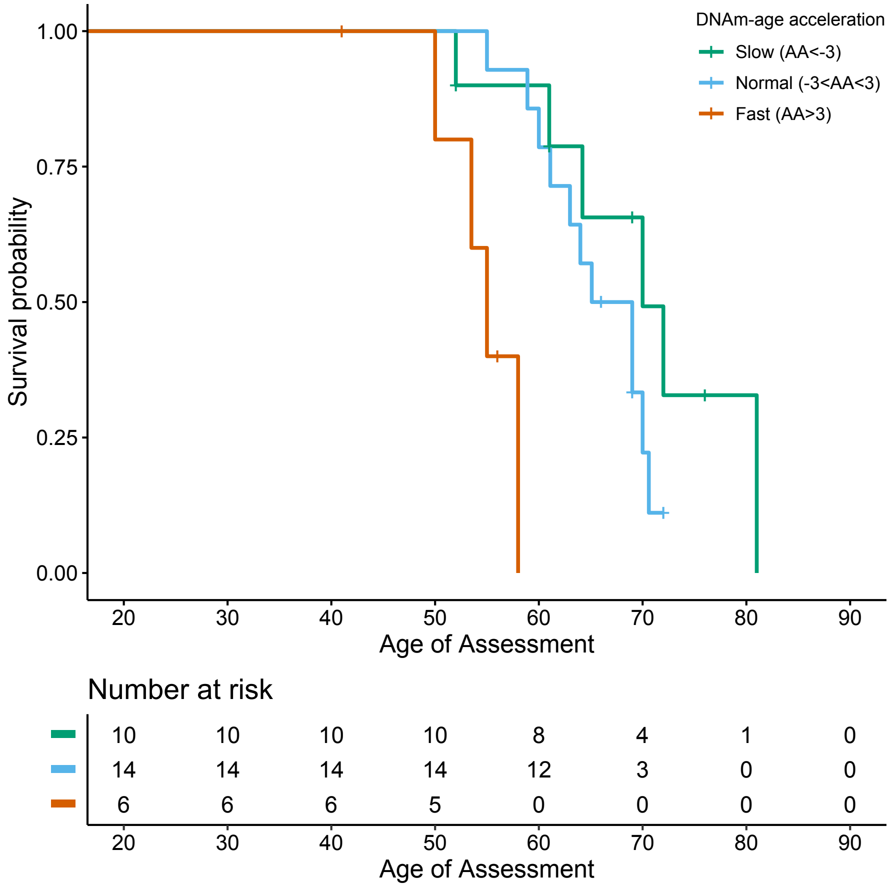

Fig. S1. Kaplan-Meier curve of disease survival probability in 30 *C9orf72* ALS patients stratified into three groups; slow aging: DNAm-age acceleration <-3 years, normal aging: DNAm-age acceleration is between -3 and 3 years, and fast aging: DNAm-age acceleration >3 years. AA represents DNAm-age acceleration.

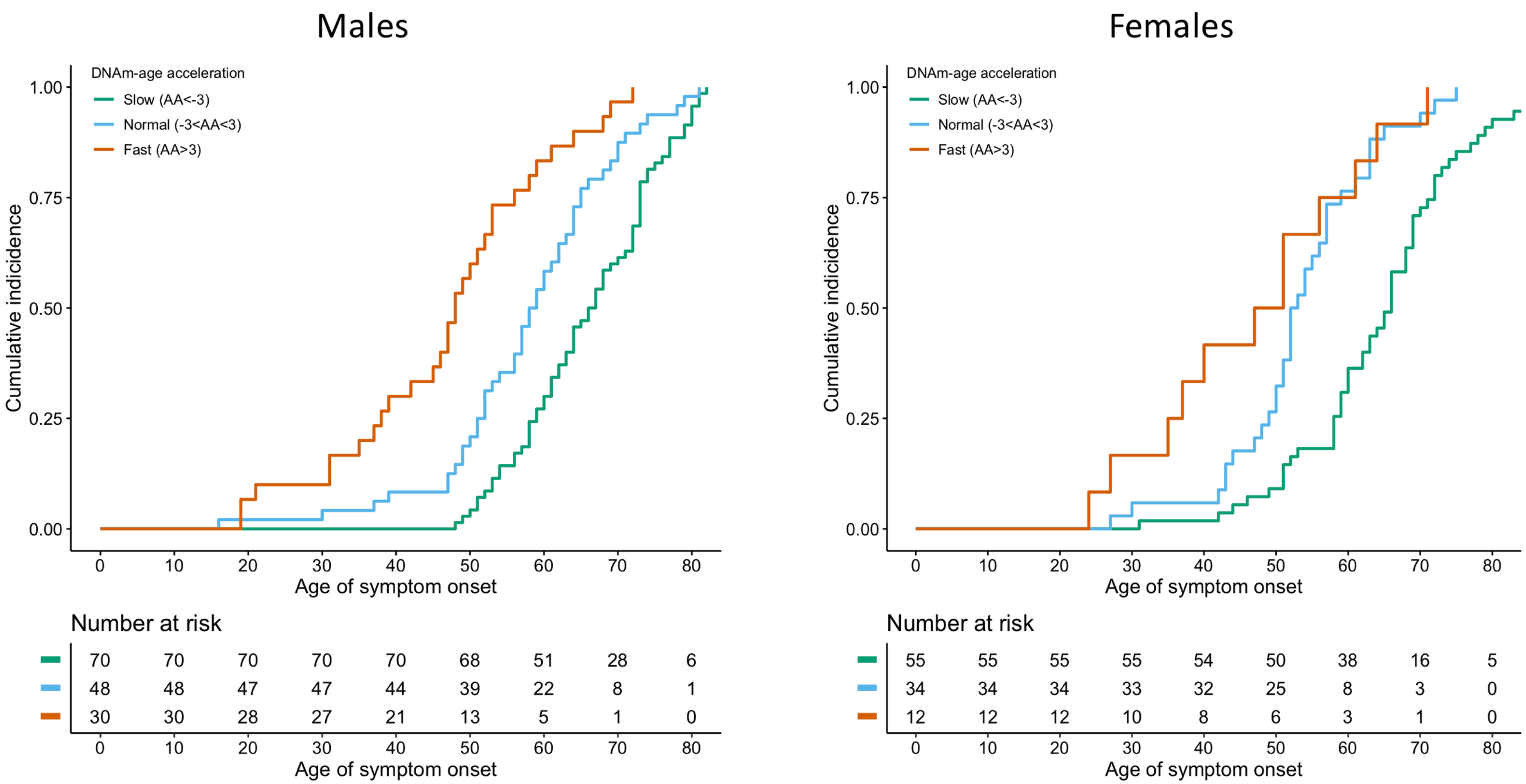

Fig. S2. Kaplan-Meier curve of cumulative incidence of disease onset in male (n=148) and female (n=101) ALS patients. AA represents DNAm-age acceleration. The association of DNAm-age acceleration with age of onset was significant in both males (p-value=2.2E-10, HR=2.09, 95%CI:1.66-2.62) and females (p-value=3E-7, HR=2.09, 95%CI:1.57-2.76).

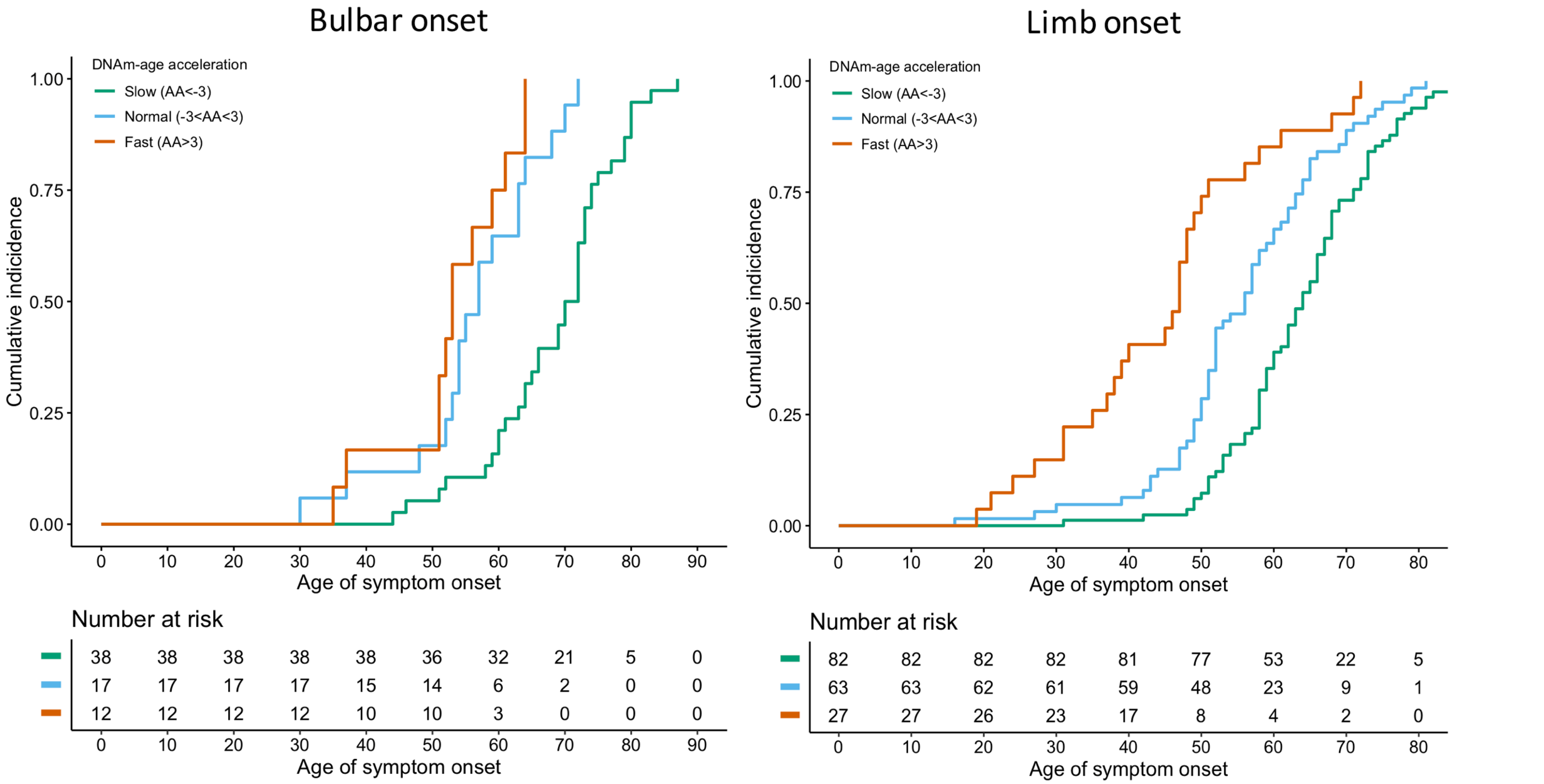

Fig. S3. Kaplan-Meier curve of cumulative incidence of disease onset in ALS patients with bulbar (n=67) and limb onset (n=172). AA represents DNAm-age acceleration. The association of DNAm-age acceleration with age of onset was significant in both bulbar onset (p-value=4.8E-8, HR=2.8,95%CI:1.93-4.05) and limb onset (p-value=1.5E-9, HR=1.96, 95%CI:1.58-2.43) ALS patients.

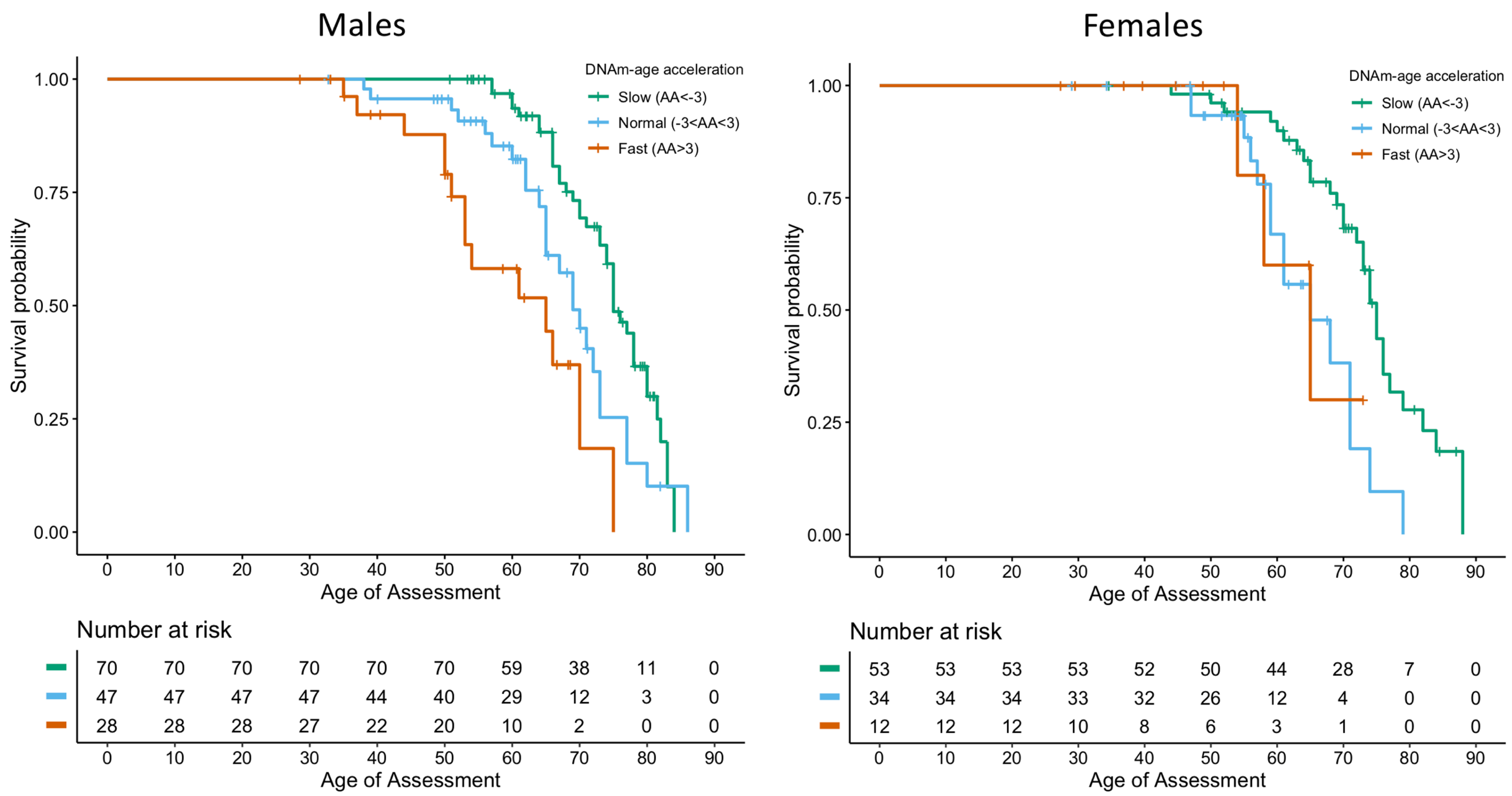

Fig. S4. Kaplan-Meier curve of survival probability in male (n=148, age of assessment for 3 cases were not available) and female (n=101, age of assessment for 2 cases was not available) ALS patients. AA represents DNAm-age acceleration. The link between DNAm-age acceleration and disease survival is significant in both males (p-value=8.4E-6, HR=2.1, 95%CI:1.5-2.9) and females (p-value=0.002, HR=2.1, 95%CI:1.3-3.3).

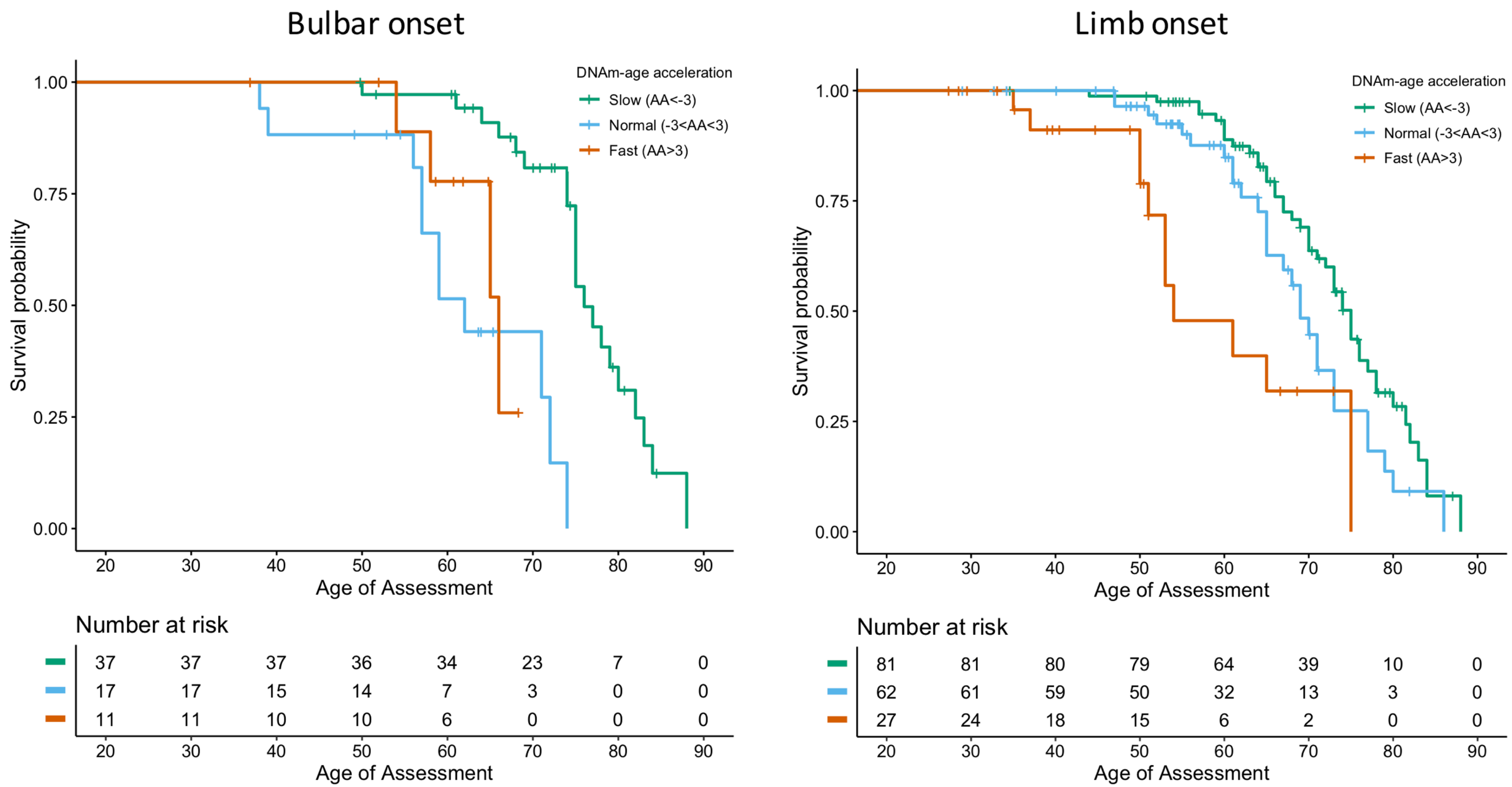

Fig. S5. Kaplan-Meier curve of survival probability in ALS patients with bulbar (n=67, age of assessment for 2 cases were not available) and limb onset (n=172, age of assessment for 2 cases were not available). AA represents DNAm-age acceleration. The link between DNAm-age acceleration and disease survival was significant in both bulbar (p-value=0.0003, HR=2.7, 95%CI:1.6-4.7) and limb onset (p-value=0.0001, HR=1.9, 95%CI:1.4-2.6) ALS patients.

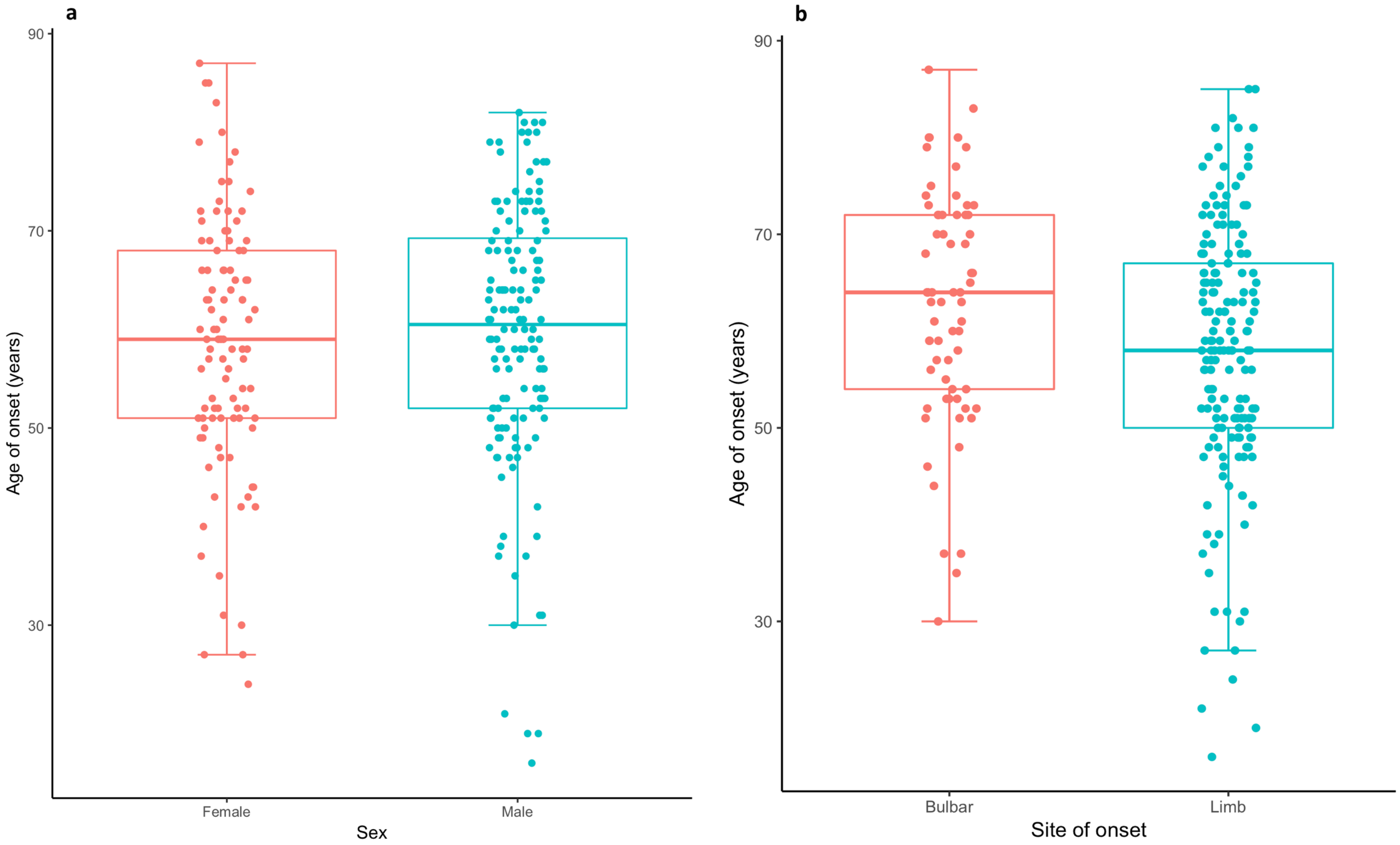

Fig. S6. Boxplot of age of onset in (a) male and female ALS patients; and (b) patients with bulbar and limb onset. The crossbar represents the median age of onset. There was no significant difference in age of onset between male and female patients (p-value=0.42, MWU test), but we observed a 6-year later age of onset in ALS patients with bulbar vs. limb onset (p-value=0.01, MWU test). After adjustment to sex and site of onset, DNAm-age acceleration is still significantly associated with age of onset (adjusted p=2E-16, B=-1.26, R^2=0.35, multivariate linear regression).

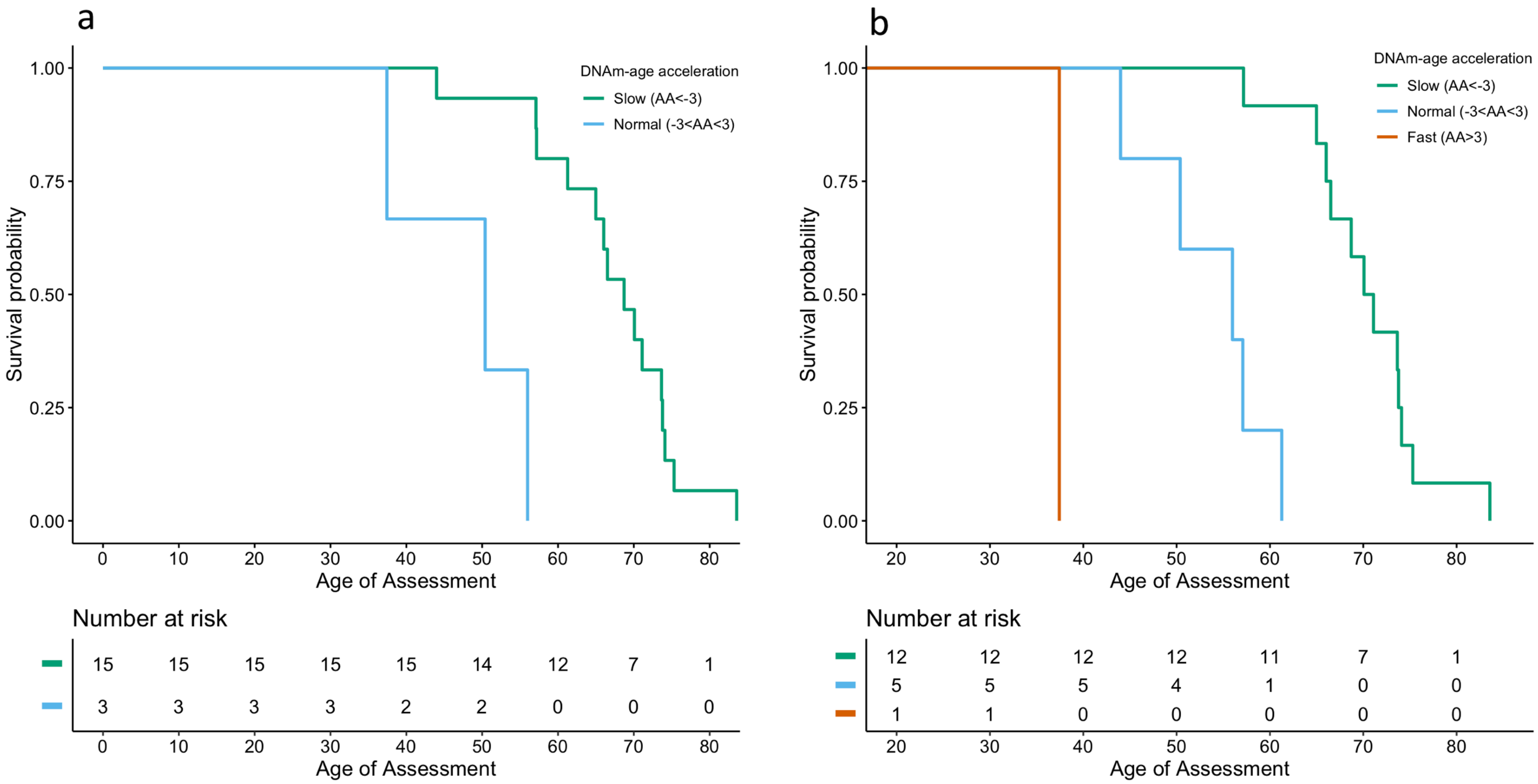

Fig. S7. Kaplan-Meier curve of survival probability in ALS patients in (a) frontal cortex or (b) cervical spinal cord tissues. Frontal cortex based DNAm-age acceleration is associated with survival in ALS patients (p=0.006, median age of survival is 69 vs 50 years in slow vs normal aging groups). Cervical spinal cord based DNAm-age acceleration is associated with survival in ALS patients (p=0.0009, median age of survival is 71 vs 37 years in slow vs fast aging groups).

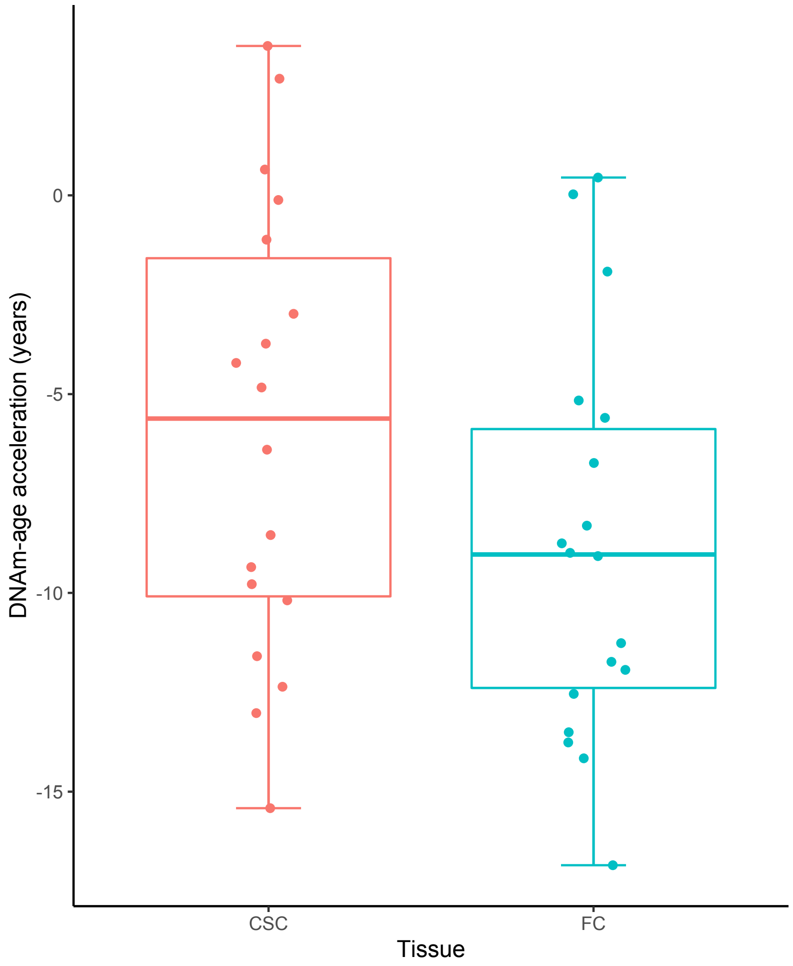

Fig. S8. Boxplot of DNAm-age acceleration in frontal cortex (FC) and cervical spinal cord (CSC) from the same ALS patients (n=18).

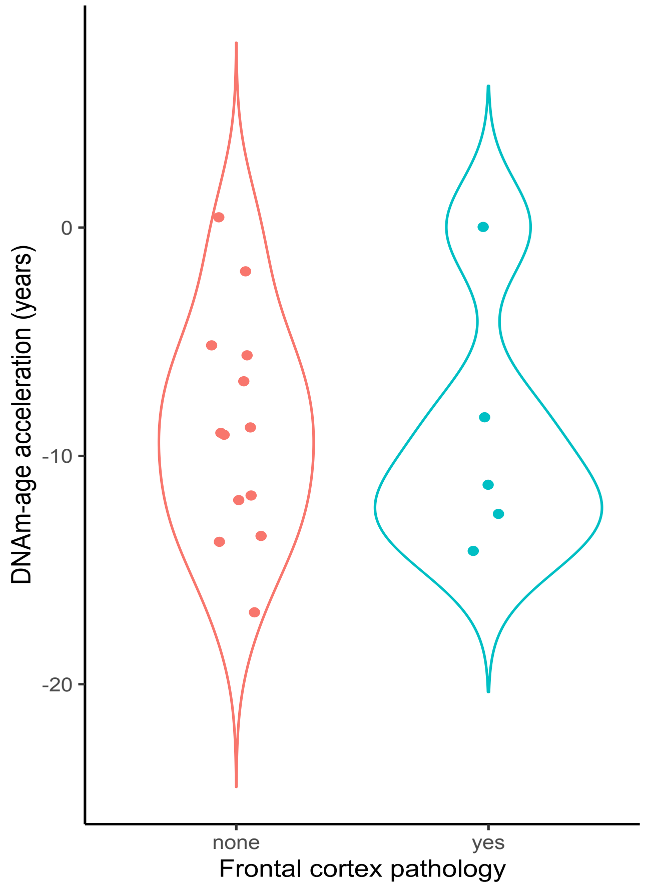

Fig. S9. Violin plot of frontal cortex DNAm-age acceleration of ALS patients with or without Frontal cortex pathology. Frontal cortex pathology is defined as tau inclusions or TDP-43 inclusions (Table S1). 5 ALS patients are with frontal cortex pathology and 13 ALS patients are free from frontal cortex pathology. No significant difference of DNAm-age acceleration was found between two groups.

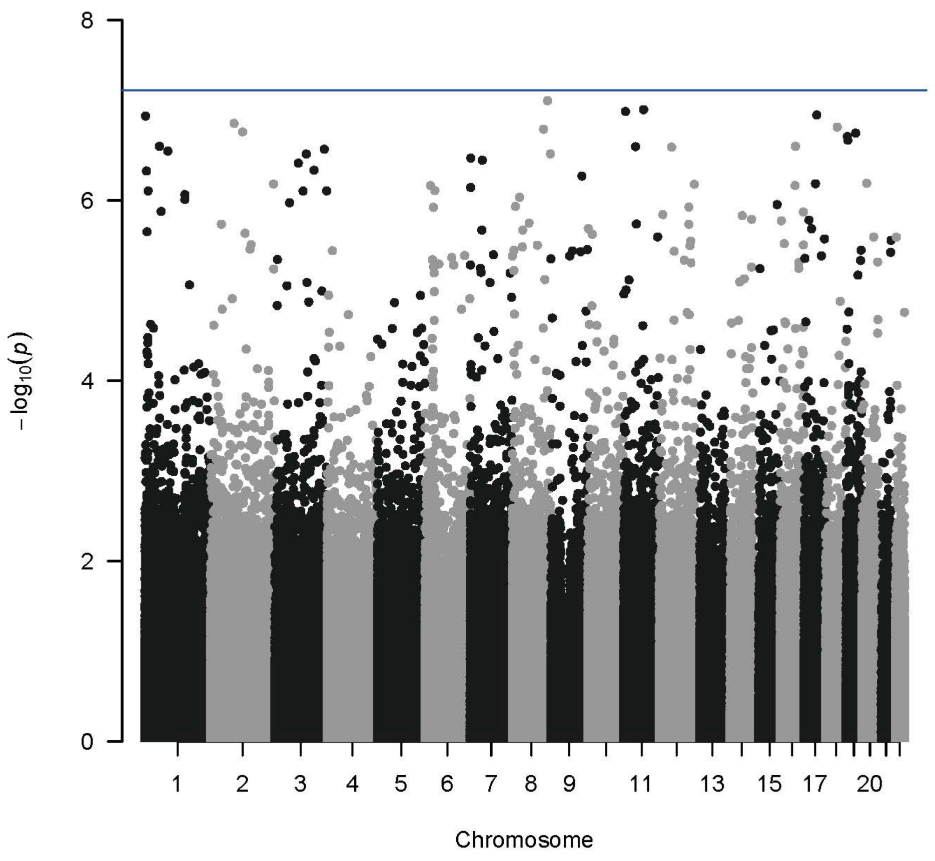

Fig. S10. Manhattan plot presenting the association between locus-by-locus DNAm status of 835,424 CpG-sites and age of onset. Multivariate linear regression analysis did not reveal any CpGs associated with age of onset at the genome-wide significant level. The blue line represents genome-wide significance (adjusted p-value=6.0E-8).
